# Iowa University

**Published:** 1856-06

**Authors:** 


### COLLEGE OF PHYSICIANS AND SURGEONS.

### IOWA UNIVERSITY.

It is a source of much satisfaction to the friends of this State enterprise, to know that the above department of the University is now in so promising and prosperous a condition. It is now so well understood as to become a common remark, and the members of the Faculty are daily enquired of with reference to its prospects, and as often congratulated upon their signal success. This is very gratifying, indeed, to them in being assured that their labors are appreciated, and that there is in the public mind so strong a sentiment in favor of the enterprise.

In the progress of their efforts they have encountered many discouragements; have suffered from the most illiberal and unjust opposition open and covert, unmanly and ungenerous; have been annoyed and molested by measures too low to find a conception in the breasts of men of elevated sentiment; and yet, amid all they have pressed forward, looking neither to the right nor the left, but kept in view the establishment of an institution in our State which is emphatically a benefit to community, and one in which the profession has full confidence, for during the recent sitting of the "State Medical Society," every one with whom we conversed spoke in the most flattering terms of its present condition, and expressed the hope that as we should have one good institution in the State, this department would continue to flourish—and by resolution pledged themselves to sustain it by their influence and efforts.

While we have such emphatic endorsements, we in return for these exhibitions of generous confidence, will continue to labor for the establishment, upon an immovable basis, of an institution which shall redound to the best interests of the profession of this State. It being the only regular organization and regarded as such at home and abroad, throughout the country, the student who enters its halls has assurance of its standing and permanency.

We have invited competent men of the profession to visit us during our session, examine our appliances, observe the method of teaching, listen to the examinations and witness operations, which have been responded to by a number of our medical brethren in and out of the State, and we are willing that their reports shall decide for us. We again solicit our brethren to visit us at any time, and will esteem it as a favor should they do so. It is their *duty* in fact, to enquire into our condition, to see for themselves, and not be influenced or biased by false representations. Attempts have been made as we have learned, to lessen the number of our class of last session. We numbered seventy-nine—the names, residences, etc., we are prepared to show, of those who composed it, and we here boldly challenge a contradiction.

We would here caution the public in relation to mistakes which have grown out of the blending of names. This is important to students, as mistakes have occurred with some who left their homes to enter this institution, but were deceived into a different impression, while attempts have been made with others. Our title stands at the head of this article and preceptors, parents, and students will bear this in mind. So soon as they arrive let them enquire for Prof. J. R. Allen, M. D., who is Dean of the faculty, and who will be pleased to extend to them a welcome.

Scholarships will be distributed to those entitled to them, as heretofore, and students will call upon their Representatives and Senators in time to secure them. Those desirous of procuring them after these have been disposed of, will please address the Dean, who will make an arrangement in their favor.

Circulars will soon be distributed, but the Catalogue will be published in due time by the President of the mother University, Mr. Dean, by order of the State Trustees. The time of the opening of the next session will be announced in the "circular."

